# Exploring equity in primary-care-based physical activity interventions using PROGRESS-Plus: a systematic review and evidence synthesis

**DOI:** 10.1186/s12966-016-0384-8

**Published:** 2016-05-20

**Authors:** S Attwood, E van Sluijs, S Sutton

**Affiliations:** Centre for Diet and Activity Research (CEDAR), MRC Epidemiology Unit, University of Cambridge School of Clinical Medicine, Box 285 Institute of Metabolic Sciences, Cambridge Biomedical Campus, Cambridge, CB2 0QQ UK; Behavioural Science Group, Primary Care Unit, Institute of Public Health, University of Cambridge, Cambridge, CB2 0SR UK

**Keywords:** Physical activity, Intervention, Socio-economic inequalities, Equity, Non-communicable diseases, Trials

## Abstract

**Background:**

Little is known about equity effects in primary care based physical activity interventions. This review explored whether differences in intervention effects are evident across indicators of social disadvantage, specified under the acronym PROGRESS-Plus (place of residence, race/ethnicity, occupation, gender, religion, education, social capital, socioeconomic status, plus age, disability and sexual orientation).

**Methods:**

Six bibliographic databases were systematically searched for randomised controlled trials (RCTs) of physical activity interventions conducted in primary care. Harvest plots were used to synthesize findings from RCTs reporting subgroup or interaction analyses examining differences in intervention effects across levels of at least one PROGRESS-Plus factor.

**Results:**

The search yielded 9052 articles, from which 173 eligible RCTs were identified. Despite PROGRESS-Plus factors being commonly measured (*N* = 171 RCTs), differential effect analyses were infrequently reported (*N* = 24 RCTs). Where reported, results of equity analyses suggest no differences in effect across levels or categories of place of residence (*N* = 1RCT), race (*N* = 4 RCTs), education (*N* = 3 RCTs), socioeconomic status (*N* = 3 RCTs), age (*N* = 16 RCTs) or disability (*N* = 2 RCTs). Mixed findings were observed for gender (*N* = 22 RCTs), with some interventions showing greater effect in men than women and others vice versa. Three RCTs examined indicators of social capital, with larger post-intervention differences in physical activity levels between trial arms found in those with higher baseline social support for exercise in one trial only. No RCTs examined differential effects by participant occupation, religion or sexual orientation.

**Conclusion:**

The majority of RCTs of physical activity interventions in primary care record sufficient information on PROGRESS-Plus factors to allow differential effects to be studied. However, very few actually report details of relevant analyses to determine which population subgroups may stand to benefit or be further disadvantaged by intervention efforts.

**Electronic supplementary material:**

The online version of this article (doi:10.1186/s12966-016-0384-8) contains supplementary material, which is available to authorized users.

## Background

Tackling health inequities, defined as systematic differences in health between groups in society experiencing different levels of social disadvantage [1], remains a priority for those attempting to improve population health through intervention. Physical activity is often targeted in interventions given its beneficial effect on a range of chronic illnesses [2]. From the perspective of health inequities, engaging in fewer beneficial health behaviours, including physical activity, is one pathway through which social disadvantage may increase the risk of disease [3]. Existing evidence suggests that groups in society with less access to power, wealth or prestige may be less likely to engage in sufficient physical activity to benefit their health [4]. For example, lower activity levels have been observed in older adults, women [5], minority ethnic groups [6] and those of lower socio-economic status [7, 8], amongst other groups [9].

Acknowledging the existence of such disparities in physical activity levels, trials have been conducted to explore ways to boost activity levels in those at greatest risk of social disadvantage. To date, work in this area has tended to target specific population subgroups in order to establish the effectiveness of appropriately tailored interventions [10, 11]. While of value in determining what works best to promote activity for these particular recipient groups, this approach has been criticised for missing the opportunity to reach far greater numbers who may be inactive, yet would not qualify to participate based on their sociodemographic profiles. Population surveys show that levels of activity remain low in most countries. Hence, intervening in specific population subgroups may not prove the most effective approach to reducing health risks associated with inactive lifestyles [12].

An alternative approach aims to encourage larger numbers of people to make small changes to their lifestyles, thereby lowering the risk profile of the population as a whole [13, 14]. For example, individuals from a range of different sociodemographic backgrounds may be targeted through scalable interventions, often delivered within the context of primary care systems. These may include provision of brief advice to increase physical activity, delivered by health care practitioners or, increasingly, digital interventions using mobile phone or internet platforms. Recipients of these interventions are often provided with generic content, regardless of their personal characteristics, preferences or needs. While this approach may theoretically be considered equitable given potential for wide reach, the possibility of interventions being selectively engaged with and eliciting differential responses exists. This may lead to a subsequent widening of inequities in physical activity levels and, possibly, in associated health outcomes [15].

The need to study this issue in greater depth has recently been highlighted by the Cochrane and Campbell Equity Methods group, who advocate the use of a guidance framework known as ‘PROGRESS-Plus’ [16]. This acronym summarises a number of social stratification factors understood to influence health opportunities, including the chance to participate in and benefit from physical activity promotion interventions (place of residence, race or ethnicity, occupation, gender, religion, education, social capital, socioeconomic status (SES), plus age, disability and sexual orientation). Clear evidence now exists that a number of these factors are influential determinants of physical activity levels, with associations reported between the amount of activity performed and characteristics of a place of residence (e.g. access to trails, walkable destinations, green spaces and safety or crime rates) [17–19], an individual’s race [20–22], occupational characteristics (e.g. supportive work policies, opportunities for active commuting, manual or non-manual work) [23], gender [24, 25], level of education or degree of health literacy [7], social capital (across various measures of this multifactorial concept, including social cohesion, participation, social network size and degree of trust) [26–28], socioeconomic status (again, as indicated by various measures, including wealth or assets, income or composite measures) [7] and age [29]. The influence of remaining PROGRESS-Plus factors (e.g. religion, disability, sexual orientation) on physical activity levels appear to have been less commonly examined.

However, evidence regarding possible differences in the effect of physical activity promotion interventions across levels or categories of the PROGRESS-Plus factors is sparse. Reviews of the physical activity intervention literature that present analyses of differential effects generally show inconclusive findings across participant age groups, genders, ethnic groups, education levels and indicators of SES [30–32]. It is notable that so far only Humphreys et al. [33] have considered differential effects across all eleven PROGRESS-Plus factors. This scoping review examined environmental and policy interventions, finding that primary studies tended to record the information necessary to explore the issue of equity, yet few actually reported the results of subgroup or interaction analyses indicating for whom interventions worked best. Where differences in effect were found in this review, majority ethnic groups appeared to benefit more from environmental and policy interventions than minority groups. Gender differences were also noted, but with no clear conclusions drawn as to which type of intervention consistently benefitted either men or women [33].

The present review extends this work by scoping the extant literature in order to summarise available evidence on potential differences in the effect of individual-level physical activity interventions across levels or groups of all eleven PROGRESS-Plus factors. By using this approach, we add to the nascent body of literature exploring health inequities by examining gradients in response to interventions, rather than focusing on disadvantaged groups only [33–35].

This review focuses on adult populations in the context of primary care, defined as a patient’s first point of contact with the medical system, with care provided by a generalist rather than specialist member of health care staff. Primary care has been chosen as the context of interest here given that it is a common setting for the delivery of preventative health and lifestyle information to a wide range of patient groups.

## Methods

### Study design

We conducted a systematic scoping review of published literature available in Pubmed (MEDLINE), CINAHL, The Cochrane Library, EMBASE, BNI and PsycINFO. Findings are presented using both narrative and graphical syntheses.

### Search strategy & inclusion criteria

Systematic searches were conducted in August 2014 and updated in March 2016 to identify published articles reporting details of randomised controlled trials (RCTs) of physical activity interventions in primary care. Search terms were chosen to reflect review inclusion and exclusion criteria (Table 1). Eligibility screening of RCT titles, abstracts and full texts was performed by the first author (SA). The remaining authors (SS, EvS) each independently screened half of all identified articles, with final decisions on eligibility based on consensus between at least two authors at all stages of the screening process. Differences in opinions regarding eligibility were resolved through discussion. Data extraction and risk of bias assessments were conducted by the first author and double checked for accuracy by remaining authors (SS, EvS). Search terms and strategies are available in Additional file 1.

**Table 1:** Review Inclusion & Exclusion Criteria

Domain	Inclusion criterion	Exclusion criterion
Population	Adults (≥16 years)	Interventions recruiting primary care staff
Intervention	Interventions targeting physical activity, fitness or sedentary behaviour, including as part of a multi-component intervention	Rehabilitation interventions (e.g. following an illness or incident), including physiotherapy or interventions to manage the side effects of a treatment regime
Design	Randomised controlled trials (RCTs) comparing intervention effect in intervention versus control groups or in two or more intervention groups	
Outcome	A post-intervention measure of physical activity, fitness or sedentary behaviour	A post-intervention measure of a psychological or other mediator of activity (i.e. intentions to be active) with no behavioural measure
Setting	Interventions recruiting participants from primary care. The intervention itself may be conducted elsewhere (e.g. exercise referral schemes).	Interventions in residential care, nursing homes or other institutionalised settings, or that included participants receiving substantial inpatient care

### Study classification and data extraction

We first classified the included RCTs based on their treatment of PROGRESS-Plus, basing our definitions of each of these factors on the work of O’Neill et al. [16] and on the measures most commonly employed within the field of physical activity research (see Additional file 2). Where a measure was classifiable under more than one PROGRESS-Plus factor (e.g. an indicator of employment status is relevant to ‘occupation’ but also to ‘socio-economic status’ (SES)), we included it under the factor deemed more appropriate.

Full data extraction wsa conducted for those RCTs that examined differential intervention effects using interaction or subgroup analyses for at least one PROGRESS-plus factor. We identified subgroup analyses if authors conducted separate significance tests of an intervention effect in each level or category of a PROGRESS-Plus factor. We identified interaction analyses if authors used an overall test to directly compare differences in intervention effects across levels or categories of a PROGRESS-Plus factor [36].

The data extraction form contained three sections: the first captured information on PROGRESS-Plus (i.e. measures used and analyses conducted), the second included the Cochrane risk of bias tool [37], and the third captured descriptive information on the trial (intervention and measurement details), plus outcome data from both main and differential effect analyses. Studies were judged to be at low, medium, high or unclear risk of bias overall based on their performance across criteria determining the risk of selection, performance, detection, attrition, reporting and other biases. For RCTs measuring at least one PROGRESS-Plus factor (but that did not conduct differential effect analyses), data was extracted on PROGRESS-Plus only. This information was classified as either being derived from within the publication text or baseline demographic characteristics tables or if any PROGRESS-Plus factor had been adjusted for as part of the main effect analysis reported in the text or main results tables.

### Data synthesis

We synthesized the differential effect analyses reported in included papers using Harvest plots [38]. In these plots, each RCT is represented by a single bar, with bar position indicating for whom the intervention was more effective. The height of the bars represents the sample size of each RCT, the number above the bar is the study reference, the colour of the bars represents the type of analysis (i.e. interaction or subgroup) informing our decisions on differential effect, and the bar pattern indicates the overall risk of bias. Where it was unclear from the published paper what type of analysis had been performed, or the direction or significance of effects, authors were contacted for clarification. In studies reporting both subgroup and interaction analyses, interaction analyses were preferentially selected to inform our decisions given that these provide a more direct test. Where multiple physical activity outcomes were given, we opted for the measure deemed least prone to error or bias (i.e. multiple-item or validated tools or objective rather than self-report measures). Data pertaining to the longest physical activity follow-up were extracted in instances where more than one time point was reported as we considered this to provide the most robust evidence for differential effects. Where differences in the results of subgroup or interaction analyses were present at earlier time points, these are noted in the Results section below.

## Results

### Search results

**Fig. 1:**
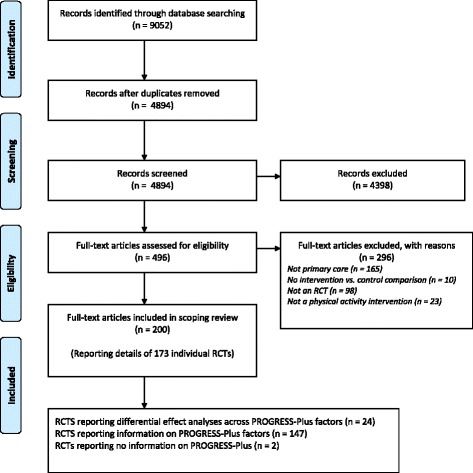
PRISMA Flow Diagram of Selection Process

Our search strategy identified 9,052 papers, of which 200 (reporting details of 173 individual RCTs) were eligible for inclusion following title, abstract and full text screening (see Additional file 3). The numbers of papers available at each stage of the screening process and reasons for exclusions are provided in Fig. 1.

### PROGRESS-Plus differential effect analyses

Of the 173 eligible RCTs, 24 reported subgroup or interaction analyses exploring intervention effects across at least one PROGRESS-Plus factor. Table 2 provides further details of these trials [39–62]. All were conducted in the context of primary care systems of developed nations. Seven focused exclusively on older populations, recruiting samples over 60 years of age. The length of study follow-up varied from three to 24-months, as did the content and intensity of interventions, mode of delivery (i.e. face-to-face, telephone or computer-based) and the health professional who delivered the intervention (i.e. physicians, nurses or exercise professionals).

**Table 2:** Randomized controlled trials included in the evidence synthesis

Author	Harvest Plot reference	Location	Sample Size	Sample characteristics	PROGRESS Plus measures	Intervention	Control	Physical activity measure	Longest Follow-up	Intervention effective overall?
Oxcheck study group (1994) [39]	1	UK	6124	35–64 year old general practice registered patients	Differential effects: gender	Health check involving nurse counselling to reduce cardiovascular disease risk.	Waiting list	Self-report: frequency of vigorous intensity activity (<1 session/month)	12 months	yes
					Measured: gender, social capital (marital status), socio-economic status (social class), age					
Activity Counseling Trial writing group (2001) [40]	2	US	874	35–75 year primary care patients, inactive, in stable health, English speaking, independent living, able to increase activity	Differential effects: gender	Intervention 1: control group intervention plus a behavioural counselling session with health educator and follow-up phone call.	Physician advice on national physical activity recommendations and information from an on-site health educator.	Self-report:7 day PAR (energy expenditure)	24 months	N/A – only gender subgroups presented
					Measured: race, occupation (employment status), social capital (marital status), socio-economic status (income), age			Objective V0_2_ Max		
						Intervention 2: intervention 1 plus continual telephone follow-up over follow-up.				
Burton et al. (1995) [41]	3	US	4195	>65 year community dwelling Medicare beneficiaries	Differential effects: disability (Quality of well-being scale)	Preventative health visit delivered by primary care physician and follow-up behavioural counselling if necessary	Pamphlet from American Association of Retired Persons	Self-report: frequency of physical activity (sedentary if <3 sessions/week)	24 months	No
					Measured: race, gender, education (years completed), social capital (marital status), socio-economic status (income), age, disability					
Carroll et al. (2010) [42]	4	US	394	Adult primary care patients, inactive, able to increase activity	Differential effects: race, gender	Computer tailored feedback based on self-reported physical activity	Computer tailored feedback report on preventative tests	Self-report:7 day PAR (energy expenditure)	6 months	No
					Measured: race, occupation (employment status), gender, education (level), social capital (marital status), socio-economic status (income), age					
Conroy et al. (2014) [43]	5	US	99	45–64 year old, female primary care patients with a BMI ≥ 25 kg/m^2^, able to increase activity	Differential effects: race	Interventionist led 12-weekly group programme incorporating activity goal setting, pedometer and activity self-tracker and mindfulness concepts	Self-guided 12-weekly programme based on the American Health Association’s ‘Choose to Move’ Programme	Self-report: Modifiable Activity Questionnaire (MAQ; MET hours/week total activity)	12 months	No
					Measured: race, education, social capital (marital status), age					
Glasgow et al. (2012) [44]	6	US	463	25–75 year primary care patients with type 2 diabetes, body mass index (BMI) > 25 kg/m^2^ and one risk factor for heart disease (high blood pressure, high cholesterol, smoker), English or Spanish speaking, able to increase activity	Differential effects: race, gender, education (level), age	Intervention 1: internet-based computer assisted diabetes self-management intervention	Enhanced usual care (computerised health risk appraisal feedback)	Self-report: CHAMPS questionnaire (energy expenditure)	12 months	Yes
					Measured: race, gender, education, social capital (chronic illness resource survey), socio-economic status (income), age					
						Intervention 2: intervention 1 plus human support				
Grandes et al. (2011) [45]	7	Spain	4317	20–80 family physician registered patients, inactive, stable health	Differential effects: gender, age	Brief physician advice and information plus individualised physical activity plan	Usual care	Self-report:7 day PAR (frequency and duration of physical activity)	24 months	No
					Measured: occupation (work situation), gender, education (level), socio-economic status (social class), age, disability (health related quality of life)					
								Objective V0_2_ Max		
Halbert et al. (2000) [46]	8	Australia	299	>60 community dwelling general practice patients, inactive, independent living, stable health, able to increase activity	Differential effects: gender	Exercise trainer session plus individualised physical activity advice and plan	Pamphlet promoting good nutrition for older adults	Self-report:7 day activity log (frequency and duration of physical activity)	12 months	Yes
					Measured: age					
								Objective: accelerometer (energy expenditure)		
Harrison et al. (2004) [47]	9	UK	545	>18 year primary care patients eligible for exercise referral schemes (inactive with risk factors for coronary heart disease)	Differential effects: gender, age	Exercise referral scheme consisting of consultation with exercise officer, written information and reduced entrance fees to a local leisure centre	Leaflets promoting physical activity for health and well-being	Self-report:7 day PAR (frequency and duration of physical activity)	12 months	No
					Measured: race, gender, age					
Harris et al., (2015) [48]	10	UK		60–74 year old, general practice registered patients, able to increase activity	Differential effects: gender, age, social capital (participating as a couple), disability (Townsend Disability score)	Pedometer, plus face-to-face consultations with practice nurse incorporating behaviour change techniques, handbook and walking plan	Usual care	Objective: accelerometer (change in average daily step count)	3 months	Yes
					Measured: race, occupation (retired), gender, education, social capital, Socio-economic status (IMD), age, disability					
Huber et al., (2015) [49]	11	US	90	18–55 year, obese (BMI ≥ 30 ≤ 39.9 ky/m^2^) primary care registered patients	Differential effects: gender	Portion control plate with instructions plus tele-coaching incorporating motivational interviewing over 3-months	Usual care	Self-report: International Physical Activity Questionnaire (IPAQ; total METs/week), 7 day PAR (kcal/day)	6 months	No
					Measured: race, occupation (working status) gender, education (level), social capital (marital status, household size), age					
Illife et al. (2015) [50]	12	UK	1256	≥65 year general practice registered patients, independent living and physically able to participate	Differential effects: gender, age	Intervention 1: Falls exercise management programme incorporating group sessions working on strength, balance and postural stability	Usual care	Self-report: CHAMPS questionnaire (meeting >150 min of MVPA per week), Phone-FITT and PASE	12 months	Yes
					Measured: place of residence (London or Nottingham), race (English first language), gender, education (completing further education), social capital (social network, social support), age, disability (self-rated health and physical function tests)					
						Intervention 2: Otago exercise programme incorporating home-based weight exercises				
Jakicic et al. (2009) [51]	13	US	4376	45–74 year old, overweight or obese (BMI ≥ 25 Kg/m^2^) primary care registered patients with type 2 diabetes Mellitus	Differential effects: race, gender, age	Intensive lifestyle intervention aiming to achieve weight loss and incorporating weekly group education sessions over 6 months and individual support thereafter up to 1 year	Diabetes support condition, incorporating 3 general group educational sessions covering topics of exercise and diet	Self-report: Harvard Alumni Study Leisure Time Physical Activity Questionnaire	12 months	Yes
					Measured: race, gender, age					
								Objective: cardio-respiratory fitness by graded treadmill exercise test		
Koelwijn van Loon et al. (2010) [52]	14	Netherlands	615	General practice adult patients eligible for cardiovascular risk management	Differential effects: gender, socio-economic status, age	Cardiovascular disease risk management with risk communication and nurse led motivational interviewing	Standard cardiovascular disease risk management with risk communication	Self-report: meeting national physical activity recommendations	12 months	No
					Measured: gender, socio-economic status, age					
Lakerveld et al. (2013) [53]	15	Netherlands	662	30 to 50 year general practice patients at risk of diabetes or cardiovascular diseases	Differential effects: gender, education (level), age	Healthy lifestyle counselling from practice nurses plus 3 monthly follow-up sessions	Brochure containing healthy lifestyle information	Self-report: AQuAA questionnaire (sedentary behaviour)	12 months	No
					Measured: gender, education (level), age					
Murphy et al. (2012) [54]	16	UK	2160	>16 year practice registered patients eligible for Exercise Referral Scheme	Differential effects: gender, socio-economic status (index of multiple deprivation), age	National exercise referral scheme delivered in leisure centres by exercise professionals	Usual care plus leaflet highlighting benefits of exercise	Self-report:7 day PAR (duration of exercise)	12 months	Yes
					Measured: race, occupation (employment status), gender, education (level), social capital (marital status), socio-economic status, age					
Norris et al. (2000) [55]	17	US	812	>30 year primary care patients registered to attend a well visit, able to increase activity, English speaking, stable health	Differential effects: gender, age	Physician counselling based on PACE protocol and written exercise prescription. Follow-up phone calls in a subset of participants	Usual care	Self-report: PASE questionnaire (physical activity score) and Paffenbarger’s physical activity index	6 months	Yes
					Measured: race, gender, education (level), social capital (marital status), age, disability (health status)					
Petrella et al. (2003) [56]	18	Canada	284	>65 year community dwelling primary care patients, not participating in formal exercise training, able to increase activity, independent living, stable health	Differential effects: gender, age	Physician administered step test plus counselling and recommendations	Usual care	Objective: V0_2_ Max	12 months	Yes
					measured: gender, education (years complete), social capital (marital status), socio-economic status (income), age					
Petrella et al. (2010) [57]	19	Canada	360	55–85 year community dwelling primary care patients, inactive, English speaking, able to increase activity, stable health	Differential effects: place of residence (urban vs. rural), gender, age	Individualised exercise prescription based on step test results, physician counselling and exercise prescription tailored to stage of change.	individualised exercise prescription based on step test results	Self-report:7 day PAR (energy expenditure)	12 months	No
								Objective V0_2_ Max		
					Measured: place of residence, occupation (employment status), gender, education (level), social capital (living status, marital status), age					
Purath et al. (2013) [58]	20	US	72	60–85 years, inactive, community dwelling primary care patients, stable health	Differential effects: gender, social capital (marital status, friend and family support to exercise), socio-economic status (income), age	Fitness test and feedback with goal setting (PACE protocol) plus 10-week telephone follow-up.	Nutrition intervention using a similar format PACE protocol	Self-report: CHAMPS questionnaire (physical activity frequency and energy expenditure)	6 months	No
								Objective: Senior fitness test (body strength, aerobic endurance and balance)		
					Measured: race, gender, education (years complete), social capital, socio-economic status, age					
van Sluijs et al. (2005) [59]	21	Netherlands	396	18–70 year primary care patients with hypertension, hyper cholesterolaemia or non-insulin dependent diabetes, inactive, able to increase activity	Differential effects: gender, age	Health care provider consultation discussing physical activity plus two PACE physical activity counsellor visits and telephone follow up	Usual care plus brief physical activity promotion	Self-report: SQUASH questionnaire (duration of physical activity and meeting recommendations)	12 months	No
					Measured: occupation (employment status), gender, education (level), age					
Steptoe et al. (2001) [60]	22	UK	883	General practice registered adult patients with high risk of cardiovascular disease, stable health	Differential effects: gender, age, education (attainment)	Nurse behavioural counselling to increase physical activity	Usual care	Self-report: Stages of Change for physical activity questionnaire	12 months	Yes
					Measured: race, occupation (employment status), gender, education (attainment), social capital (marital status, social support), age					
Stewart et al. (2001) [61]	23	US	173	65–90 year inactive Medicare health maintenance organisation enrolees, stable health, able to increase activity, English speaking	Differential effects: gender, age	Individually tailored programme encouraging participation in community classes plus optional group workshops	Waiting list	Self-report: CHAMPS questionnaire (energy expenditure)	12 months	Yes
					Measured: race, occupation (working status), gender, education (level), social capital (marital status), socio-economic status (income), age, disability (self-rated health)					
van Steenkiste et al. (2007) [62]	24	Netherlands	490	40–75 year general practice registered patients at risk of cardiovascular disease	Differential effects: gender	Physician consultations using a decision support tool encouraging lifestyle change.	Physician consultation with standard written cholesterol guidelines	Self-report: duration of physical activity (>2 h/week)	6 months	Yes
					Measured: gender, socio-economic status (level), age					

Self-report measures of physical activity were most commonly used (*N* = 22 RCTs). Where objective measures were employed (*N* = 8 RCTs), maximum oxygen uptake (VO_2_ max), a proxy measure of physical fitness, was used in five RCTs, physical activity measured using accelerometers in two and submaximal metabolic equivalent of tasks (METS) reported in one RCT. The main effect of the intervention trialled in 11 RCTs was reported as null. 12 trials observed a significant group difference favouring the intervention arm (one RCT reported only effect estimates disaggregated by gender). We summarise information on differential effects derived from all RCTs regardless of the results of main effect because subgroup differences in response may be present even when a significant main effect is not found. We judged five of the 24 RCTs to be at low risk of bias, 19 to be at medium risk of bias and none to be at high risk of bias.

### PROGRESS-Plus evidence synthesis

**Fig. 2:**
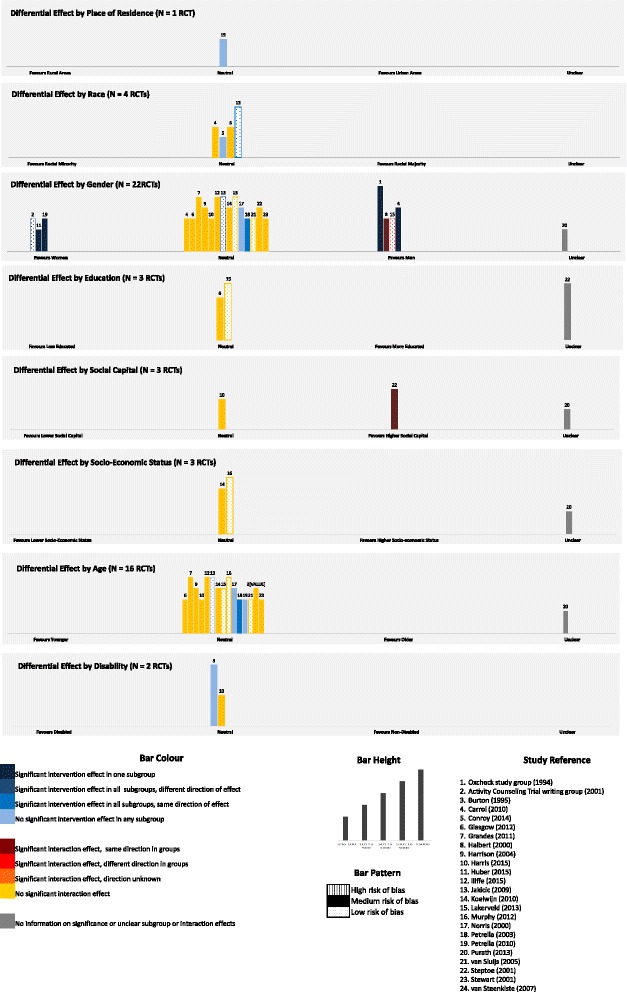
PROGRESS-Plus Evidence Synthesis

**Fig. 3:**
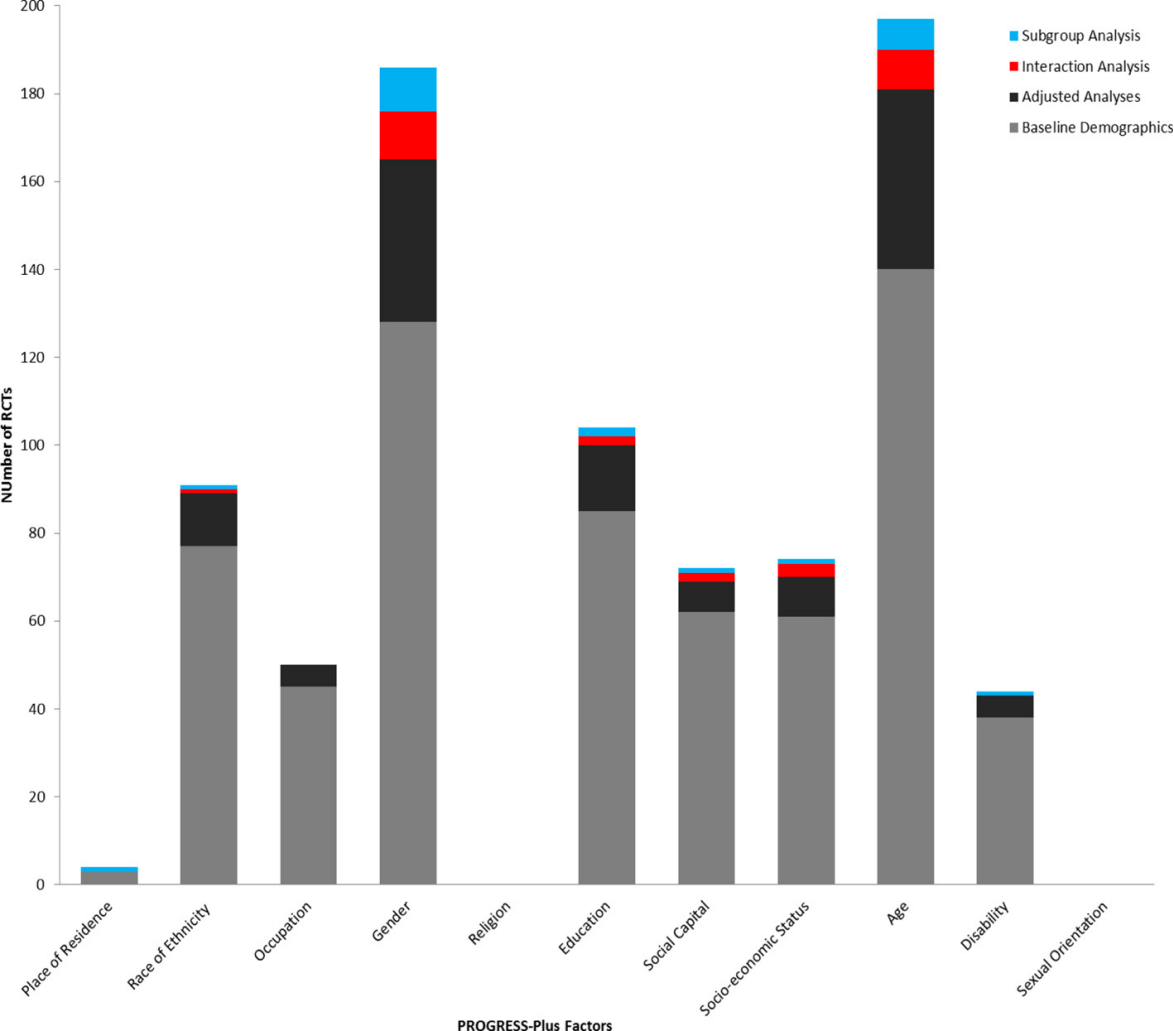
PROGRESS-Plus reporting in included RCTs. Legend: Bars sum to greater than 173 as RCTs may report PROGRESS-Plus factors in more than one way

Figure 2 presents the differential effects evidence. Just one RCT examined trial outcomes across subgroups of place of residence and two RCTs across indicators of disability (one subgroup analysis, one interaction analysis). This is despite *N* = 41 RCTs measuring disability and five reporting details of place or residence in baseline demographic tables or adjusting for these factors in main effect analyses (Fig. 3). No significant differences in intervention effects were seen in participants residing in either urban or rural settings [56], or in those with either poor versus good self-rated health [41] or with no versus any disability based on Townsend Disability Score [48].

Three RCTs each examined interaction terms between trial arms and the PROGRESS-Plus factors education, social capital and socio-economic status (from *N* = 101, *N* = 70 and *N* = 68 reporting on these factors, respectively) (Fig. 3). Across these three factors, we were unable to clarify results of analyses presented in two RCTs, and were unsuccessful in our attempts to obtain further information from the authors [58, 60]. For education, there was no strong indication that interventions produced different outcomes for participants who had completed more versus less than high/secondary school education [44, 53], although we note that Lakerveld et al. [53], found a significant trial arm by education interaction at interim 12-month follow-up (intervention effects favoured participants with higher education levels). This effect was not sustained at 24-months. For social capital, one RCT examined the indicator of social support, finding significantly larger post-intervention differences in physical activity between trial arms in those with higher baseline social support for exercise [60]. A second RCT explored differences in effect according to whether participation was as an individual or as a couple (randomisation by household), finding a borderline non-significant result (*p* < 0.06, greater effect in those participating as a couple) at 3-month follow-up [48]. For socio-economic status, no differences in intervention effect were reported in one RCT employing the Welsh Index of Multiple Deprivation [54], or another that classified participant socio-economic status as low, intermediate or high (measure unreported) [52].

Four RCTs reported differential effect analyses by participant race (from *N* = 88 RCTs that measured this characteristic) (Fig. 3). There was no evidence of differences in intervention effect between participants who were either Latino versus non-Latino [44] or white versus black [42] based on interaction analyses, nor between white versus non-white female participants (African American, Asian or ‘other’) [43] or across five ethnicity classifications based on subgroup analysis [51]. However, Conroy et al. [43] did identify a significant subgroup effect at an earlier (3-month) follow-up, showing a significant intervention effect in white women.

A larger number of RCTs examined differential effects by age and gender, although these still comprised only a relatively small percentage of all RCTs to record information on these factors (*N* = 22 RCTs of 150 RCTs measuring gender, and *N* = 16 RCTs of *N* = 161 RCTs measuring age; Fig. 3). For gender, eight subgroup analyses and 14 interaction analyses compared intervention effects between male and female participants. We were unable to clarify the results of analyses conducted in one RCT [58]. A small majority of trials (*N* = 14) found no differential effects by gender. Where differential effects were reported, these were mainly identified through subgroup analyses: there were two instances of interventions proving effective in men only [39, 42], and three where the inverse was true [40, 49, 57]. One interaction analysis demonstrated a greater intervention effect in men, although it was also effective for women [46], while another showed a significant interaction effect favouring a reduction in sedentary time in men, but not in women at 24-month follow-up [53]. Sample sizes for RCTs demonstrating differential effects by gender were generally between 101 and 500 randomised participants, with the exception of that conducted by the Oxcheck study group [39]. This larger trial found effects favouring men, based on a comparison of 952/1770 men and 1184/2218 women in intervention/control groups, respectively. Otherwise, there appeared to be no consistent pattern across the type of intervention, length of follow-up, sample characteristics, significance of the trial’s main effect or the extent of risk of bias in RCTs showing differential effects in opposing directions.

For age, four subgroup and 11 interaction analyses were available (of *N* = 161 RCTs to measure participant age). Where authors provided details of the age groupings used in their analyses, dichotomous comparisons were based on the threshold values of over and under 50 years [45], 55 years [55], 65 years [50, 57], 70 years [56] and 75 years [61]. Of these, four RCTs also recruited older adults only (over 55 years of age) [50, 56, 57, 61]. One RCT compared participants aged 16–44 years with those between 45–59 and over 60 years [54], one presents five-year age brackets (60–64, 65–69, 70–75 years) [48] and another 10-year age brackets (45–55, 56–65, 66–76 years) [51], while the remainder treated age as a continuous variable. No differential intervention effects were apparent in any RCT reporting interaction and subgroup analyses by age, although the RCT conducted by Grandes et al., [45] did identify a significant interaction effect at an earlier six-month follow-up (showing a significant intervention effect in participants ≥50 years, but no effect in those <50 years). This was not sustained through to 24-month follow-up [45]. We were unable to clarify the type of analysis conducted in one trial [58].

No RCTs explored differential effects by participant occupation and none of the 173 trials included in this review either measured or examined differential effects by religion or sexual orientation (Fig. 3). Two RCTs provided no information on any PROGRESS-Plus factors [63, 64].

## Discussion

### Main findings

The aims of this review were to scope the existing literature in order to summarise how PROGRESS-Plus factors are reported in published RCTs of physical activity interventions conducted in primary care and to synthesise information on differences in intervention effects across levels or groups of these social stratifiers, where available. Our results indicate that only a small number of RCTs report relevant analyses that allow us to draw conclusions regarding the differential effectiveness of physical activity interventions in this context. Where available, interaction and subgroup analyses tended to be limited to age and gender, with no evidence of differences in response across categories of the former, and mixed evidence regarding the latter, with some interventions more effective in men and others more effective in women. It is likely that features unique to each RCT, such as the type of intervention (e.g. content, setting or mode of delivery), the sample recruited (e.g. age, location or SES) or trial methods (i.e. the outcome measures used) are responsible for the mixed effects observed for gender. Unfortunately, there are too few available RCTs to determine which, if any, of these factors consistently lead to larger post-intervention increases in physical activity in either men or women.

For the remaining PROGRESS-Plus factors, neutral effects were found for all except social capital, with one RCT showing that higher social capital, conceptualised as greater social support for exercise, increased the likelihood of improvements in physical activity levels following the intervention. A second RCT measuring this factor found no significant differences in effect. To clarify this finding, further work exploring the impact of social support, and indeed other indicators of social capital, on physical activity intervention effectiveness is now indicated.

In general, reporting of differential effect analyses was sparse despite the fact that the majority of included RCTs collected sufficient information on PROGRESS-Plus to permit study of intervention equity across at least one of the factors included in this acronym. Encouraging researchers to familiarise themselves with the PRISMA-Equity 2012 Extension reporting checklist [65] may prompt more frequent consideration of the issue of equity when reporting results of trials of physical activity interventions. Although specifically relevant to the reporting of systematic reviews and meta-analyses, this checklist does highlight where in the intervention process inequities may occur and how to analyse and report differences in intervention effects across the PROGRESS-Plus factors. We welcome ongoing development of equity reporting guidelines specific to different types of study design (e.g. the CONSORT-equity guideline to improve reporting of health equity in randomized trials) [66].

Given the importance of examining differential effects of interventions, we reiterate here recommendations to continue to conduct and report subgroup and interaction analyses. This is despite recognised limitations in terms of power, given that studies rarely aim to recruit enough participants to examine differences between any groups other than trial arms [67]. However, we do draw attention to criteria that authors are advised to consider when determining the ‘credibility’ of results obtained from these types of analyses [36]. These specify ‘good practice’, such as defining the subgroup or interaction effects to be studied a priori (i.e. in a study protocol) and conducting analyses based on a hypothesised logic model. Where subgroup and interaction analyses are exploratory, it is important to state this to ensure that the results are not over-interpreted.

Applying these ‘good practice’ criteria to the present review, we note that authors of only one of the 24 RCTs included in our evidence synthesis stated that their study was powered to detect an intervention versus control group difference across categories of a PROGRESS-Plus factor [40]. In this case, participant gender was examined, finding effects that favoured men. Fifteen of 24 RCTs chose to pre-specify which subgroup or interaction analyses they would conduct prior to reporting the results (i.e. in Methods sections or protocol papers), whereas just four provided justifications for why such analyses were to be performed or referenced evidence suggesting that differences in effect were to be expected based on an identified mechanism. For the remaining nine RCTs included in the evidence synthesis, it was unclear how many post-hoc subgroup or interaction analyses were conducted for every one reported in the published paper. Improvements in how differential effect analyses are conducted and reported on are now required if the evidence base in this area is to develop.

### Strengths and limitations of the evidence base

This review highlights where gaps in the physical activity intervention literature exist in relation to equity. The evidence base has significant limitations. None of the included RCTs measured religion or sexual orientation. This is despite evidence to indicate that these factors may influence physical activity [68, 69]. Where such omissions remain, we are unable to determine the impact of primary care based physical activity interventions for all groups in society at risk of disadvantage.

As with all reviews, we are limited by the quality and comprehensiveness of the reporting of primary studies. We acknowledge that meta-analysing data on intervention effects across PROGRESS-Plus factors would have been useful, but were prevented from doing so both by incomplete reporting of subgroup and interaction analyses within included RCTs and by the sparsity of data available for most PROGRESS-Plus factors. Future systematic reviews exploring the issue of equity may attempt to contact all original trial authors in order extract, combine and re-analyse data across PROGRESS-Plus from all included RCTs using individual participant data meta-analysis. However, we do recognise that this work is likely to be impeded by difficulties in operationalizing PROGRESS-Plus factors, especially those that represent multi-faceted concepts that are defined and measured in diverse ways. Considering SES for example, both individual and area-level measures of this concept exist, each captured by multiple indicators. This makes it very difficult to compare data from different trials that conceptualise PROGRESS-Plus factors differently. The choice of measure used to study a PROGRESS-Plus factor is also likely to influence whether differences in effect are able to be identified and which hypotheses are put forward to explain how such effects may occur.

To help overcome these issues, we recommend that further work is conducted to define each factor in the context of a given health behaviour. Specifying the logic models theorised to underlie differences in intervention effects according to each PROGRESS-Plus factor, using both qualitative investigation and moderated-mediation analysis techniques, may help in this endeavour [70, 71]. Such work would permit the evidence base on the equity effects of health interventions to develop in a more systematic manner and would facilitate synthesis of data from individual trials by encouraging use of the same PROGRESS-Plus measures, thereby overcoming the issue of low power reducing the change of detecting true subgroup differences.

One further limitation of the present review is that we have not directly considered how uptake of the physical activity interventions included in our evidence synthesis may have differed according to PROGRESS-Plus factors, and how such differences impact subsequent conclusions regarding equity. While a comprehensive summary of differences in trial uptake according to PROGRESS-Plus is beyond the scope of this review, we note that many of the included RCTs employed relatively strict participant recruitment criteria (e.g. they may have excluded potential participants with contraindications to exercise, thereby omitting some disabled groups and often, older individuals). Although generally implemented for legitimate reasons, the use of exclusive recruitment criteria limits the heterogeneity of study samples, so restricting exploration of equity effects to studying somewhat homogenous samples. For example, considering the PROGRESS-Plus factor of age, threshold values were frequently used to classify individuals into dichotomous age groups for differential effect analyses, with cut-points often set at older ages (e.g. over or under 75 years). Where age restrictions were also applied at the point of recruiting individuals into a trial (e.g. RCTs focussing on older adults only), there may then have been too little variation between age groups analysed to allow for contrasting effects of an intervention to be clearly identified.

## Conclusion

In summary, the majority of RCTs of individual level physical activity interventions in primary care record sufficient information on PROGRESS-Plus factors to allow potential differences in effect to studied. However, very few actually report the details of relevant analyses to enable us to determine which population subgroups may stand to benefit or be further disadvantaged by intervention efforts. There is currently too little evidence to enable us to draw firm conclusions regarding the impact of physical activity interventions on the health equity of recipients.

## Additional files

Additional file 1: Database Search Terms & Strategies. (DOC 75 kb) Additional file 2: Definitions of PROGRESS-Plus factors. (DOCX 15 kb) Additional file 3: Scoping Review Eligible Randomised Controlled Trials (RCTs) (DOC 195 kb)
