# Supplementary material for: Exploring equity in primary-care-based physical activity interventions using PROGRESS-Plus: a systematic review and evidence synthesis
Source: Int J Behav Nutr Phys Act. 2016 May 20;13:60. doi: 10.1186/s12966-016-0384-8 (PMC4875625; doi:10.1186/s12966-016-0384-8)
Supplement: Additional file 1: — Database Search Terms & Strategies. (DOC 75 kb) [file 12966_2016_384_MOESM1_ESM.doc]

**Additional File 1: Database Search Terms & Strategies**

| **Inclusion Criteria** | **Search Terms** |
| --- | --- |
| Intervention | Counselling OR counseling OR intervention OR ‘health promotion’ OR ‘health education’ OR ‘behaviour modification’ OR ‘behavior modification’ OR advice OR  ‘patient education’ OR ‘health communication’ |
| Setting | ‘primary health care’ OR ‘primary care’ OR ‘family practice’ OR ‘general practice’ OR ‘family physician’ OR ‘family doctor’ OR ‘practice nurse’ OR GP OR ‘health care’ OR ‘primary medical care’ |
| Outcome | exercise OR ‘physical education’ OR sport OR walk OR jog OR run OR swim OR ‘physical fitness’ OR cycling OR dance OR ‘physical activity’ OR ‘exercise referral’ OR ‘active lifestyle’ OR sedentary |
| Design | RCT OR trial OR random |

*Source: NICE Healthcare Databases (EMBASE, MEDLINE, PsychINFO, BNI, CINAHL)**

|  | 1 | EMBASE, MEDLINE, PsycINFO, BNI, CINAHL | (counselling OR counseling OR intervention OR "health promotion" OR "health education" OR "behaviour modification" OR "behavior modification" OR advice OR "patient education" OR "health communication").ti,ab | [1300706](http://www.library.nhs.uk/hdas/search-results/1?PageNumber=1&PageSize=10&SortBy=srt.unspecified&ShowAbstracts=False&BackToSearchResultsCount=1&databases=bnj.pqt.bnia&databases=bnj.ebs.cinahl&databases=bnj.ovi.emez&databases=bnj.ovi.prmz&databases=bnj.ovi.psyh) |
| --- | --- | --- | --- | --- |
|  | 2 | EMBASE, MEDLINE, PsycINFO, BNI, CINAHL | ("primary health care" OR "primary care" OR "family practice" OR "general practice" OR "family physician" OR "family doctor" OR "practice nurse" OR GP OR "health care" OR "primary medical care").ti,ab. | [982102](http://www.library.nhs.uk/hdas/search-results/2?PageNumber=1&PageSize=10&SortBy=srt.unspecified&ShowAbstracts=False&BackToSearchResultsCount=1&databases=bnj.pqt.bnia&databases=bnj.ebs.cinahl&databases=bnj.ovi.emez&databases=bnj.ovi.prmz&databases=bnj.ovi.psyh) |
|  | 3 | EMBASE, MEDLINE, PsycINFO, BNI, CINAHL | (exercise OR "physical education" OR sport* OR walk* OR jog* OR run* OR swim* OR "physical fitness" OR cycling OR dance OR "physical activity" OR "exercise referral" OR "active lifestyle" OR sedentary).ti,ab | [1299533](http://www.library.nhs.uk/hdas/search-results/3?PageNumber=1&PageSize=10&SortBy=srt.unspecified&ShowAbstracts=False&BackToSearchResultsCount=1&databases=bnj.pqt.bnia&databases=bnj.ebs.cinahl&databases=bnj.ovi.emez&databases=bnj.ovi.prmz&databases=bnj.ovi.psyh) |
|  | 4 | EMBASE, MEDLINE, PsycINFO, BNI, CINAHL | (RCT OR trial OR random*).ti,ab | [2320245](http://www.library.nhs.uk/hdas/search-results/4?PageNumber=1&PageSize=10&SortBy=srt.unspecified&ShowAbstracts=False&BackToSearchResultsCount=1&databases=bnj.pqt.bnia&databases=bnj.ebs.cinahl&databases=bnj.ovi.emez&databases=bnj.ovi.prmz&databases=bnj.ovi.psyh) |
|  | 5 | EMBASE, MEDLINE, PsycINFO, BNI, CINAHL | 1 AND 2 AND 3 AND 4 | [3430](http://www.library.nhs.uk/hdas/search-results/5?PageNumber=1&PageSize=10&SortBy=srt.unspecified&ShowAbstracts=False&BackToSearchResultsCount=1&databases=bnj.pqt.bnia&databases=bnj.ebs.cinahl&databases=bnj.ovi.emez&databases=bnj.ovi.prmz&databases=bnj.ovi.psyh) |

*Source: The Cochrane Library**

|  | 1 | Cochrane Library | (counselling OR counseling OR intervention OR "health promotion" OR "health education" OR "behaviour modification" OR "behavior modification" OR advice OR "patient education" OR "health communication").ti,ab | [87266](http://www.library.nhs.uk/hdas/search-results/1?PageNumber=1&PageSize=10&SortBy=srt.unspecified&ShowAbstracts=False&BackToSearchResultsCount=1&databases=bnj.pqt.bnia&databases=bnj.ebs.cinahl&databases=bnj.ovi.emez&databases=bnj.ovi.prmz&databases=bnj.ovi.psyh) |
| --- | --- | --- | --- | --- |
|  | 2 | Cochrane Library | ("primary health care" OR "primary care" OR "family practice" OR "general practice" OR "family physician" OR "family doctor" OR "practice nurse" OR GP OR "health care" OR "primary medical care").ti,ab. | [82609](http://www.library.nhs.uk/hdas/search-results/2?PageNumber=1&PageSize=10&SortBy=srt.unspecified&ShowAbstracts=False&BackToSearchResultsCount=1&databases=bnj.pqt.bnia&databases=bnj.ebs.cinahl&databases=bnj.ovi.emez&databases=bnj.ovi.prmz&databases=bnj.ovi.psyh) |
|  | 3 | Cochrane Library | (exercise OR "physical education" OR sport* OR walk* OR jog* OR run* OR swim* OR "physical fitness" OR cycling OR dance OR "physical activity" OR "exercise referral" OR "active lifestyle" OR sedentary).ti,ab | [125788](http://www.library.nhs.uk/hdas/search-results/3?PageNumber=1&PageSize=10&SortBy=srt.unspecified&ShowAbstracts=False&BackToSearchResultsCount=1&databases=bnj.pqt.bnia&databases=bnj.ebs.cinahl&databases=bnj.ovi.emez&databases=bnj.ovi.prmz&databases=bnj.ovi.psyh) |
|  | 4 | Cochrane Library | #1 and #2 and #3 (in Trials) | [3540](http://www.library.nhs.uk/hdas/search-results/4?PageNumber=1&PageSize=10&SortBy=srt.unspecified&ShowAbstracts=False&BackToSearchResultsCount=1&databases=bnj.pqt.bnia&databases=bnj.ebs.cinahl&databases=bnj.ovi.emez&databases=bnj.ovi.prmz&databases=bnj.ovi.psyh) |

*original search conducted in August 2014. An updated search was performed in March 2016 covering the period from August 2014 to March 2016 using an identical search strategy and search terms
