# Supplementary material for: Exploring equity in primary-care-based physical activity interventions using PROGRESS-Plus: a systematic review and evidence synthesis
Source: Int J Behav Nutr Phys Act. 2016 May 20;13:60. doi: 10.1186/s12966-016-0384-8 (PMC4875625; doi:10.1186/s12966-016-0384-8)
Supplement: Additional file 2: — Definitions of PROGRESS-Plus factors. (DOCX 15 kb) [file 12966_2016_384_MOESM2_ESM.docx]

**Additional File 2: Definitions of PROGRESS-Plus factors***

| **PROGESS - Plus Factor** | **Description** | **Example of measures** |
| --- | --- | --- |
| Place of residence | Locations in which individuals reside or perceptions of their location. | E.g. urban/rural/ inner-city classifications, perceptions of the local environment (e.g. the Neighbourhood Environment Walkability Scale)[1] |
| Race | Self-identified racial or ethnic group or other classifications of culture or language. This includes nationality status (e.g. refugee or migrant). | E.g. racial or ethnic group classifications (white/mixed or multiple ethnicity/ asian/black/other), mother tongue or country of origin |
| Occupation | Occupational situation, patterns of work or features of the working environment | E.g. unemployed/employed/retired, manual or non-manual work, full-time or part-time employment, graded hierarchies measuring occupational status or prestige |
| Gender | Sex refers to the biological and physiological characteristics that differentiate men and women.  Self-identified gender incorporates ideas around socially constructed roles or differences in behaviour attributed to men and women[2] | E.g. male or female classifications |
| Religion | An individuals’ religious affiliation or system of religious or spiritual beliefs or values[3] | E.g. Self-reported religious denomination, details of belief systems or values held, religious activities engaged in or attendance at religious institutions[4] |
| Education | Self-reported extent and type of schooling, education or other formal training or learning undertaken | E.g. number of years in full-time education, educational attainment or qualifications achieved, institutions attended (e.g. high school/some college/college graduate/university) |
| Social capital | A multifaceted concept capturing the obligations and benefits conferred upon an individual by their society and social relationships. This can be seen as a measure of interconnectedness between an individual and their social surroundings or group[5] | E.g. perceptions of social norms surrounding trust or reciprocity, social support (e.g. marital or living status, household size, social support from friends, family or relevant others), social networks, civic participation or group membership. |
| Socio-economic status | An individual’s position within a hierarchical social structure[6]. Measures of socio-economic status aim to capture access to resources, privilege, power or control. | E.g. poverty level, income (continuous level or proportion falling into income brackets), asset-based measures such as car ownership or housing tenure, receipt of MEDICAID or other forms of state welfare or health payment assistance |
| Age | Self-reported age in years | E.g. mean or median age of a study sample or proportions falling in age brackets |
| Disability | A term incorporating ideas surrounding impairment, activity limitation and restrictions on ability to participate in certain life situations. Disability can be both mental and physical.[7]  *NB: medical contraindications to exercise or other health conditions are not considered as a disability unless authors specifically note that such conditions are functionally limiting, disability or causing impairment.* | E.g. measures of functioning, health status or quality of life (e.g. EuroQol or the SF-36),[8] physical tests of function (e.g. the walk test), or other indicators or disability (e.g. wheelchair bound) |
| Sexual orientation | Self-reported sex towards which an individual feels attraction or self-defined sexual identity[9] | E.g. hetero- /homo- /bi- /transsexual classifications |

^*We acknowledge that the definitions and measures within this table are not exhaustive and include it here to provide an example of the type of data sought and extracted.^

**References**

1. Cerin E, Saelens BE, Sallis JF, et al. Neighborhood Environment Walkability Scale: validity and development of a short form. *Med Sci Sports Exerc* 2006;**38**(9):1682-1691.

2. Word Health Organisation. http://www.euro.who.int/en/health-topics/health-determinants/gender/gender-definitions (2002). *Accessed 9 Jun 201*5.

3. Koenig HG. Religion, spirituality, and health: the research and clinical implications. *ISRN psychiatry* 2012;278730.

4. Idler EL, Musick MA, Ellison CG, et al. Measuring Multiple Dimensions of Religion and Spirituality for Health Research: Conceptual Background and Findings from the 1998 General Social Survey. *Res Aging* 2003;**25**(4):327-365.

5. Harpham T, Grant E, Thomas E. Measuring social capital within health surveys: key issues. *Health Policy Plan* 2002; **17**(1):106-111.

6. Braveman PA, Cubbin C, Egerter S et al. Socioeconomic status in health research: one size does not fit all. *JAMA* 2005;**294**(22):2879-2888.

7. World Health Organisation. http://www.who.int/topics/disabilities/en/ (2015). *Accessed 9 Jun 2015*.

8. Cohen ME, Marino RJ. The tools of disability outcomes research functional status measures. *Arch Phys Med Rehab*. 2000; **81**:S21-S29.

9. Sell R. Defining and Measuring Sexual Orientation: A Review. *Arch Sex Behav* 1997;**26**(6):643-658.
