# Supplementary material for: Exploring equity in primary-care-based physical activity interventions using PROGRESS-Plus: a systematic review and evidence synthesis
Source: Int J Behav Nutr Phys Act. 2016 May 20;13:60. doi: 10.1186/s12966-016-0384-8 (PMC4875625; doi:10.1186/s12966-016-0384-8)
Supplement: Additional file 3: — Scoping Review Eligible Randomised Controlled Trials (RCTs) (DOC 195 kb) [file 12966_2016_384_MOESM3_ESM.doc]

**Additional File 3: Scoping Review Eligible Randomised Controlled Trials (RCTs)**

| **Number** | | **Review Reference** |
| --- | --- | --- |
| **RCTs Reporting Differential Effect Analyses** | | |
| 1 | | Imperial Cancer Research Fund OXCHECK Study Group. Effectiveness of health checks conducted by nurses in primary care: final results of the OXCHECK study. BMJ. 1995; 310: 1099-1104 |
| 2 | | Writing Group for the Activity Counseling Trial Research Group. Effects of physical activity counseling in primary care: the Activity Counseling Trial: a randomized controlled trial. Jama. 2001; 286: 677-687 |
| 3 | | Burton LC1, Paglia MJ, German PS, Shapiro S, Damiano AM. The effect among older persons of a general preventive visit on three health behaviors: smoking, excessive alcohol drinking, and sedentary lifestyle. The Medicare Preventive Services Research Team. Preventive Medicine. 1995; 24: 492-497 |
| 4 | | Carroll JK1, Lewis BA, Marcus BH, Lehman EB, Shaffer ML, Sciamanna CN. Computerized tailored physical activity reports: A randomized controlled trial. American journal of preventive medicine. 2010; 39(2): 148-156. |
| 5 | | Conroy M, Sward K, Spadaro K, Tudorascu D, Karpov I, Jones BJ, Kriska AM, Kapoor W (2015). "Effectiveness of a physical activity and weight loss intervention for middle-aged women: healthy bodies, healthy hearts randomized trial." Journal of General Internal Medicine 30(2): 207. |
| 6 | | Glasgow RE, Kurz D, King D, Dickman JM, Faber AJ, Halterman E, Woolley T, Toobert DJ, Strycker LA, Estabrooks PA, Osuna D, Ritzwoller D. Twelve-month outcomes of an Internet-based diabetes self-management support program. Patient Education and Counseling. 2012; 87(1): 81. |
| 7 | | Grandes G1, Sanchez A, Montoya I, Ortega Sanchez-Pinilla R, Torcal J, PEPAF Group. Two-year longitudinal analysis of a cluster randomized trial of physical activity promotion by general practitioners. PLoS ONE. 2011; e18363 DOI: 10.1371/journal.pone.0018363 |
| 8 | | Halbert JA, Silagy CA, Finucane PM, Withers RT, Hamdorf PA. Physical activity and cardiovascular risk factors: effect of advice from an exercise specialist in Australian general practice. Medical Journal of Australia. 2000; 173: 84-87 |
| 9 | | Harrison RA, Roberts C, Elton PJ. Does primary care referral to an exercise programme increase physical activity 1 year later? A randomized controlled trial. Journal of Public Health. 2005; 27(1): 25-33. |
| 10 | | Harris T, Kerry S, Victor C, Ekelund U, Woodcock A, Iliffe S, Whincup P, Beighton C, Ussher M, Limb E, David L, Brewin D, Adams F, Rogers A, Cook DG (2015). A primary care nurse-delivered walking intervention in older adults: PACE (pedometer accelerometer consultation evaluation)-Lift cluster randomised controlled trial. PLoS medicine 12(2). |
| 11 | | Huber J., Shapiro J., Wieland M., Croghan I., Vickers Douglas K., Schroeder D., Hathaway J., Ebbert, J.(2015) Telecoaching plus a portion control plate for weight care management: A randomized trial. Trials 16, DOI: 10.1186/s13063-015-0880-1 |
| 12 | | Iliffe S. Kendrick D., Morris R., Griffin M., Haworth D., Carpenter H., Masud T., Skelton D., Dinan-Young S., Bowling A., Gage., on behalf of the ProAct65+ research team (2015). Promoting physical activity in older people in general practice: ProAct65+ cluster randomised controlled trial. British Journal of General Practice 65(640): e731-e738. |
| 13 | | JM Jakicic, SA Jaramillo, A Balasubramanyam, B Bancroft, JM Curtis, A Mathews, M Pereira,  JG Regensteiner and PM Ribisl, Look AHEAD Study Group. Effect of a lifestyle intervention on change in  cardiorespiratory fitness in adults with type 2 diabetes: results from the Look AHEAD Study. International Journal of Obesity (2009) 33, 305–316 |
| 14 | | Koelewijn-van Loon MS, van der Weijden T, Ronda G, van Steenkiste B, Winkens B, Elwyn G, Grol R. Improving lifestyle and risk perception through patient involvement in nurse-led cardiovascular risk management: a cluster-randomized controlled trial in primary care. Preventive Medicine. 2010; 50: 35-44 DOI: 0.1016/j.ypmed.2009.11.007 |
| 15 | | Lakerveld J, Bot SD, Chinapaw MJ, van Tulder MW, Kostense PJ, Dekker JM, Nijpels G. Motivational interviewing and problem solving treatment to reduce type 2 diabetes and cardiovascular disease risk in real life: a randomized controlled trial. International Journal of Behavioral Nutrition and Physical Activity. 2013; 10: 47 DOI: 10.1186/1479-5868-10-47 |
| 16 | | Murphy SM, Edwards RT, Williams N, Raisanen L, Moore G, Linck P, Hounsome N, Din NU, Moore L. An evaluation of the effectiveness and cost effectiveness of the National Exercise Referral Scheme in Wales, UK: a randomised controlled trial of a public health policy initiative. Journal of Epidemiology and Community Health. 2012: 66; 745-753 DOI: 10.1136/jech-2011-200689 |
| 17 | | Norris SL, Grothaus LC, Buchner DM, Pratt M. Effectiveness of physician-based assessment and counseling for exercise in a staff model HMO. Preventive Medicine. 2000; 30: 513-523 DOI: 10.1006/pmed.2000.0673 |
| 18 | | Petrella RJ1, Koval JJ, Cunningham DA, Paterson DH. Can primary care doctors prescribe exercise to improve fitness? The Step Test Exercise Prescription (STEP) project. American journal of preventive medicine. 2003; 24: 316-322 |
| 19 | | Petrella RJ, Lattanzio CN, Shapiro S, Overend T. Improving aerobic fitness in older adults: effects of a physician-based exercise counseling and prescription program. Canadian Family Physician. 2010; 56(5): e191-NaN. |
| 20 | | Purath J, Keller CS, McPherson S, Ainsworth B. A randomized controlled trial of an office-based physical activity and physical fitness intervention for older adults. Geriatric Nursing. 2013; 34(3): 204-211. |
| 21 | | van Sluijs EM1, van Poppel MN, Twisk JW, van Mechelen W. Physical activity measurements affected participants' behavior in a randomized controlled trial. Journal of Clinical Epidemiology. 2006; 59: 404-411 DOI: 10.1016/j.jclinepi.2005.08.016 |
| 22 | | Steptoe A1, Kerry S, Rink E, Hilton S. The impact of behavioral counseling on stage of change in fat intake, physical activity, and cigarette smoking in adults at increased risk of coronary heart disease. American Journal of Public Health. 2001; 91(2): 265-270. |
| 23 | | Stewart AL, Verboncoeur CJ, McLellan BY, Gillis DE, Rush S, Mills KM, King AC, Ritter P, Brown BW Jr, Bortz WM. Physical activity outcomes of CHAMPS II: a physical activity promotion program for older adults. Journals of gerontology. Series A, Biological sciences and medical sciences. 2001; 56: M465-470 |
| 24 | | van Steenkiste B1, van der Weijden T, Stoffers HE, Kester AD, Timmermans DR, Grol R. Improving cardiovascular risk management: a randomized, controlled trial on the effect of a decision support tool for patients and physicians. European Journal of Cardiovascular Prevention and Rehabilitation. 2007; 14: 44-50 DOI: 10.1097/01.hjr.0000239475.71805.1e |
| **RCTs Measuring PROGRESS Plus factors only** | | |
| 25 | | Anderson DR, Christison-Lagay J, Villagra V, Liu H, Dziura J. Managing the space between visits: a randomized trial of disease management for diabetes in a community health center. Journal of General Internal Medicine. 2001; 25: 1116-1122 |
| 26 | | Ariza Copado C, Gavara Palomar V, Muñoz Ureña A, Aguera Mengual F, Soto Martínez M, Lorca Serralta JR. Improvement of control in subjects with type 2 diabetes after a joint intervention: Diabetes education and physical activity. Atencion Primaria. 2011; 43(8): 398-406. |
| 27 | | Armit CM, Brown WJ, Marshall AL, Ritchie CB, Trost SG, Green A, Bauman AE. Randomized trial of three strategies to promote physical activity in general practice. Preventive Medicine. 2009; 48: 156-163 DOI: 10.1016/j.ypmed.2008.11.009 |
| 28 | | Armit CM, Brown WJ, Ritchie CB, Trost SG. Promoting physical activity to older adults: A preliminary evaluation of three general practice-based strategies. Journal of Science and Medicine in Sport. 2005; 8(4): 446-450. |
| 29 | | Avram, C., et al. (2011). "Dietary and physical activity counseling in high-risk asymptomatic patients with metabolic syndrome - A primary care intervention." Journal of Food, Agriculture and Environment 9(3-4): 16-19. |
| 30 | | Babazono, A., et al. (2007) Patient-motivated prevention of lifestyle-related disease in Japan: A randomized, controlled clinical trial. Disease Management and Health Outcomes 15, 119-126 DOI: 10.2165/00115677-200715020-00007 |
| 31 | | Barrera, M., et al. (2006) Social support and social-ecological resources as mediators of lifestyle intervention effects for type 2 diabetes. Journal of Health Psychology 11, 483-495 DOI: 10.1177/1359105306063321 |
| 32 | | Bóveda-Fontán, J., et al. (2015). "Effectiveness of motivational interviewing in patients with dyslipidemia: a randomized cluster trial." BMC family practice 16(1): 151. |
| 33 | | Blanchard, C. M., et al. (2007). "Explaining physical activity levels from a self-efficacy perspective: The physical activity counseling trial." Annals of Behavioral Medicine 34(3): 323-328. |
| 34 | | Bull, F. C., et al. (1999) Effects of tailored, personalized and general health messages on physical activity. Patient Education and Counseling 36, 181-192 |
| 35 | | Butler, C., et al. (2013). "Training practitioners to deliver opportunistic multiple behaviour change counselling in primary care: a cluster randomised trial." BMJ (Clinical Research Edition) 346(7901). |
| 36 | | Campbell, N. C., et al. (1998) Secondary prevention in coronary heart disease: a randomised trial of nurse led clinics in primary care. Heart (British Cardiac Society) 80, 447-452 |
| 37 | | Chalder, M., et al. (2012). "A pragmatic randomised controlled trial to evaluate the cost-effectiveness of a physical activity intervention as a treatment for depression: the treating depression with physical activity (TREAD) trial." Health Technology Assessment 16(10): 1-167. |
| 38 | | Chiu, C. W. and F. K. Wong (2010) Effects of 8 weeks sustained follow-up after a nurse consultation on hypertension: a randomised trial. International Journal of Nursing Studies 47, 1374-1382 DOI: 10.1016/j.ijnurstu.2010.03.018 |
| 39 | | Christian, J. G., et al. (2008). "Clinic-based support to help overweight patients with type 2 diabetes increase physical activity and lose weight." Archives of Internal Medicine 168(2): 141-146. |
| 40 | | Cocker, K., et al. (2012) Web-based, computer-tailored, pedometer-based physical activity advice: development, dissemination through general practice, acceptability, and preliminary efficacy in a randomized controlled trial. Journal of medical Internet research 14, e53 DOI: 10.2196/jmir.1959 |
| 41 | | Cohen, L. B., et al. (2011) Pharmacist-led shared medical appointments for multiple cardiovascular risk reduction in patients with type 2 diabetes. Diabetes Educator 37, 801-812 DOI: 10.1177/0145721711423980 |
| 42 | | Craft, L. L., et al. (2007) Intervention study of exercise for depressive symptoms in women. Journal of women's health (2002) 16, 1499-1509 DOI: 10.1089/jwh.2007.0483 |
| 43 | | Croteau, A., et al. (2014). "Efficacy of Using Physical Activity Mentors to Increase the Daily Steps of Older Adults in the Primary Care Setting: A Pilot Study." Journal of Aging & Physical Activity 22(1): 16-25. |
| 44 | | Cupples, M. E. and A. McKnight (1994) Randomised controlled trial of health promotion in general practice for patients at high cardiovascular risk. BMJ (Clinical research ed.) 309, 993-996 |
| 45 | | Daley, A., et al. (2008) Feasibility of an exercise intervention for women with postnatal depression: a pilot randomised controlled trial. British Journal of General Practice 58, 178-183 |
| 46 | | Davies, M. J., et al. (2008). "Effectiveness of the diabetes education and self management for ongoing and newly diagnosed (DESMOND) programme for people with newly diagnosed type 2 diabetes: Cluster randomised controlled trial." BMJ 336(7642): 491-495. |
| 47 | | Davies, M., et al. (2015). "A community-based primary prevention programme for type 2 diabetes in the UK: A cluster randomised controlled trial." Diabetes 64. |
| 48 | | Dawes, D., et al. (2014). Preventing diabetes in primary care: A feasibility cluster randomized trial." Canadian Journal of Diabetes 38. |
| 49 | | De, K., et al. (2011). "Increasing physical activity in Belgian type 2 diabetes patients: a three-arm randomized controlled trial." International Journal of Behavioral Medicine 18(3): 188-198. |
| 50 | | Deakin, T. A., et al. (2006) Structured patient education: the diabetes X-PERT Programme makes a difference. Diabetic Medicine 23, 944-954 DOI: 10.1111/j.1464-5491.2006.01906.x |
| 51 | | Dickinson, W. P., et al. (2013). "Use of a website to accomplish health behavior change: If you build it, will they come? And will it work if they do?" Journal of the American Board of Family Medicine 26(2): 168-176. |
| 52 | | Dubbert, P. M., et al. (2002) Effects of nurse counseling on walking for exercise in elderly primary care patients. Journals of gerontology. Series A, Biological sciences and medical sciences 57, M733-740 |
| 53 | | Duda, J. L., et al. (2014). "Effects of a standard provision versus an autonomy supportive exercise referral programme on physical activity, quality of life and well-being indicators: A cluster randomised controlled trial." International Journal of Behavioral Nutrition and Physical Activity 11(1). |
| 54 | | Dutton, G. R., et al. (2007) Promoting physical activity for low-income minority women in primary care. American Journal of Health Behavior 31, 622-631 DOI: 10.5555/ajhb.2007.31.6.622 |
| 55 | | Eakin, E., et al. (2009). "Telephone Counseling for Physical Activity and Diet in Primary Care Patients." American journal of preventive medicine 36(2): 142-149. |
| 56 | | Eakin, E. G., et al. (2007) Resources for health: a primary-care-based diet and physical activity intervention targeting urban Latinos with multiple chronic conditions. Health Psychology 26, 392-400 DOI: 10.1037/0278-6133.26.4.392 |
| 57 | | Eakin. E. et al. (2014). Living Well With Diabetes: 24-Month Outcomes From a Randomized Trial of Telephone-Delivered Weight Loss and Physical Activity Intervention to Improve Glycemic Control. Diabetes Care 37(8): 2177-2186. |
| 58 | | Edelman, D., et al. (2015). Nurse-led behavioral management of diabetes and hypertension in community practices: a randomized trial." Journal of General Internal Medicine 30(5): 626. |
| 59 | | Elley, C., et al. (2003). "Effectiveness of counselling patients on physical activity in general practice: cluster randomised controlled trial." BMJ (Clinical Research Edition) 326(7393): 793. |
| 60 | | Ely, A. C., et al. (2008) Kansas primary care weighs in: a pilot randomized trial of a chronic care model program for obesity in 3 rural Kansas primary care practices. Journal of rural health 24, 125-132 DOI: 10.1111/j.1748-0361.2008.00148.x |
| 61 | | Eriksson, K. M., et al. (2006) A randomized trial of lifestyle intervention in primary healthcare for the modification of cardiovascular risk factors. Scandinavian Journal of Public Health 34, 453-461 DOI: 10.1080/14034940500489826 |
| 62 | | Ferrer, R. L., et al. (2009). "A medical assistant-based program to promote healthy behaviors in primary care." Annals of Family Medicine 7(6): 504-512. |
| 63 | | Fuller, N. R., et al. (2014). "Changes in physical activity during a weight loss intervention and follow-up: A randomized controlled trial." Clinical Obesity 4(3): 127-135. |
| 64 | | Gao, S., et al. (2016). Physical activity counseling in overweight and obese primary care patients: Outcomes of the VA-STRIDE randomized controlled trial. Preventive Medicine Reports 3: 113-120. |
| 65 | | Glasgow, R. E., et al. (2006) Robustness of a computer-assisted diabetes self-management intervention across patient characteristics, healthcare settings, and intervention staff. American Journal of Managed Care 12, 137-145 |
| 66 | | Glynn, L. G., et al. (2014) Effectiveness of a smartphone application to promote physical activity in primary care: The SMART MOVE randomised controlled trial. British Journal of General Practice 64, e384-e391 DOI: 10.3399/bjgp14X680461 |
| 67 | | Goldstein, M. G., et al. (1999) Physician-based physical activity counseling for middle-aged and older adults: a randomized trial. Annals of Behavioral Medicine 21, 40-47 DOI: 10.1007/BF02895032 |
| 68 | | Gomez-Huelgas, R., et al. (2015). Effects of a long-term lifestyle intervention program with Mediterranean diet and exercise for the management of patients with metabolic syndrome in a primary care setting. European journal of internal medicine 26(5): 317. |
| 69 | | Graham-Clarke, P. and B. Oldenburg (1994) The effectiveness of a general-practice-based physical activity intervention on patient physical activity status. Behav. Change 11, 132-144 |
| 70 | | Greaves, C., et al. (2015). Waste the waist: A pilot randomised controlled trial of a primary care based intervention to support lifestyle change in people with high cardiovascular risk. International Journal of Behavioral Nutrition and Physical Activity 12(1). |
| 71 | | Green, B. B., et al. (2002) Effectiveness of telephone support in increasing physical activity levels in primary care patients. American journal of preventive medicine 22, 177-183 |
| 72 | | Greenwood, A., et al. (2015). Overcoming Clinical Inertia: A Randomized Clinical Trial of a Telehealth Remote Monitoring Intervention Using Paired Glucose Testing in Adults With Type 2 Diabetes. Journal of Medical Internet Research 17(7). |
| 73 | | Griffin, J., et al. (2014). Multiple behaviour change intervention and outcomes in recently diagnosed type 2 diabetes: the ADDITION-Plus randomised controlled trial. Diabetologia 57(7): 1308. |
| 74 | | Haber, D. and M. G. Lacy (1993) Evaluation of a socio-behavioral intervention for changing health behaviors of older adults. Behavior, Health and Aging 3, 73-85 |
| 75 | | Halbert, J., et al. (2001) Primary care-based physical activity programs: effectiveness in sedentary older patients with osteoarthritis symptoms. Arthritis and Rheumatism 45, 228-234 DOI: 10.1002/1529-0131(200106)45:3<228::AID-ART253>3.0.CO |
| 76 | | Hardcastle, S., et al. (2008). "A randomised controlled trial on the effectiveness of a primary health care based counselling intervention on physical activity, diet and CHD risk factors." Patient Education & Counseling 70(1): 31-40. |
| 77 | | Harland, J., et al. (1999) The Newcastle exercise project: a randomised controlled trial of methods to promote physical activity in primary care. BMJ (Clinical research ed.) 319, 828-832 |
| 78 | | Harris, M. F., et al. (2012). "A cluster randomised controlled trial of vascular risk factor management in general practice." Medical Journal of Australia 197(7): 387-393. |
| 79 | | Hasandokht, T., et al. (2015). Lifestyle interventions for hypertension treatment among Iranian women in primary health-care settings: Results of a randomized controlled trial. Journal of research in medical sciences : the official journal of Isfahan University of Medical Sciences 20(1): 54. |
| 80 | | Heinrich, E., et al. (2010). "Effect evaluation of a Motivational Interviewing based counselling strategy in diabetes care." Diabetes Research & Clinical Practice 90(3): 270-279. |
| 81 | | Heron, N., et al. (2014). Steps to a better Belfast: physical activity assessment and promotion in primary care. British Journal of Sports Medicine 48(21): 1558-1564. |
| 82 | | Hesselink, E., et al. (2015). Effects of a lifestyle program in subjects with Impaired Fasting Glucose, a pragmatic cluster-randomized controlled trial. BMC family practice 16(1): 183. |
| 83 | | Hillsdon, M., et al. (2002) Advising people to take more exercise is ineffective: a randomized controlled trial of physical activity promotion in primary care. International journal of epidemiology 31, 808-815 |
| 84 | | Hoseini, H., et al. (2014). Investigating the effect of an education plan based on the health belief model on the physical activity of women who are at risk for hypertension. Iranian journal of nursing and midwifery research 19(6): 647. |
| 85 | | Hyman, D. J., et al. (2007) Simultaneous vs sequential counseling for multiple behavior change. Archives of Internal Medicine 167, 1152-1158 DOI: 10.1001/archinte.167.11.1152 |
| 86 | | Isaacs, A., et al. (2007). "Exercise Evaluation Randomised Trial (EXERT): a randomised trial comparing GP referral for leisure centre-based exercise, community-based walking and advice only." Health Technology Assessment 11(10): 1. |
| 87 | | Jansink, R., et al. (2013). "No identifiable Hb1Ac or lifestyle change after a comprehensive diabetes programme including motivational interviewing: a cluster randomised trial." Scandinavian Journal of Primary Health Care 31(2): 119-127. |
| 88 | | Jimmy, G. and B. W. Martin (2005) Implementation and effectiveness of a primary care based physical activity counselling scheme. Patient Education and Counseling 56, 323-331 DOI: 10.1016/j.pec.2004.03.006 |
| 89 | | Jiryaee, N., et al. (2015). Comparing of goal setting strategy with group education method to increase physical activity level: A randomized trial. Journal of Research in Medical Sciences 20(10): 987-993. |
| 90 | | Jolly, K., et al. (2011). "A randomised controlled trial to compare a range of commercial or primary care led weight reduction programmes with a minimal intervention control for weight loss in obesity: The lighten up trial." Obesity Reviews 12. |
| 91 | | Katz, D. L., et al. (2008). "Impact of an educational intervention on internal medicine residents' physical activity counselling: The Pressure System Model." Journal of Evaluation in Clinical Practice 14(2): 294-299. |
| 92 | | Kelly, R. B. (1988) Controlled trial of a time-efficient method of health promotion. American journal of preventive medicine 4, 200-207 |
| 93 | | Kerse, N., et al. (2010). "Home-based activity program for older people with depressive symptoms: DeLLITE--a randomized controlled trial." Annals of Family Medicine 8(3): 214-224. |
| 94 | | Kerse, N. M., et al. (1999) Improving the health behaviours of elderly people: randomised controlled trial of a general practice education programme. BMJ (Clinical research ed.) 319, 683-687 |
| 95 | | Ketola, E., et al. (2001) Individualised multifactorial lifestyle intervention trial for high-risk cardiovascular patients in primary care. British Journal of General Practice 51, 291-294 |
| 96 | | Keyserling, T. C., et al. (2014) A comparison of live counseling with a web-based lifestyle and medication intervention to reduce coronary heart disease risk: a randomized clinical trial. JAMA internal medicine 174, 1144-1157 DOI: 10.1001/jamainternmed.2014.1984 |
| 97 | | King, D. K., et al. (2006). "Outcomes of a multifaceted physical activity regimen as part of a diabetes self-management intervention." Annals of Behavioral Medicine 31(2): 128-137. |
| 98 | | Kinmonth, A. L., et al. (2008). "Efficacy of a theory-based behavioural intervention to increase physical activity in an at-risk group in primary care (ProActive UK): a randomised trial." Lancet 371(9606): 41-49. |
| 99 | | Kirkman, M. S., et al. (1994) A telephone-delivered intervention for patients with NIDDM. Effect on coronary risk factors. Diabetes Care 17, 840-846 |
| 100 | | Kolt, G. S., et al. (2012) Healthy Steps trial: pedometer-based advice and physical activity for low-active older adults. Annals of Family Medicine 10, 206-212 DOI: 10.1370/afm.1345 |
| 101 | | Kolt, G. S., et al. (2007). "Effect of telephone counseling on physical activity for low-active older people in primary care: a randomized, controlled trial." Journal of the American Geriatrics Society 55(7): 986-993. |
| 102 | | Kreuter, M. W., et al. (2000) How does physician advice influence patient behavior? Evidence for a priming effect. Archives of family medicine 9, 426-433 |
| 102 | | Kreuter, M. W. and V. J. Strecher (1996) Do tailored behavior change messages enhance the effectiveness of health risk appraisal? Results from a randomized trial. Health Education Research 11, 97-105 |
| 104 | | Kypri, K. and H. M. McAnally (2005) Randomized controlled trial of a web-based primary care intervention for multiple health risk behaviors. Preventive Medicine 41, 761-766 DOI: 10.1016/j.ypmed.2005.07.010 |
| 105 | | Laing et al. (2014). Effectiveness of a Smartphone Application for Weight Loss Compared With Usual Care in Overweight Primary Care Patients. Ann Intern Med. 161:S5-S12. doi:10.7326/M13-3005 |
| 106 | | Lamb, S. E., et al. (2002). "Can lay-led walking programmes increase physical activity in middle aged adults? A randomised controlled trial." Journal of Epidemiology & Community Health 56(4): 246-253. |
| 107 | | Lawler, S. P., et al. (2010). "Multiple health behavior changes and co-variation in a telephone counseling trial." Annals of behavioral medicine : a publication of the Society of Behavioral Medicine 39(3): 250-257. |
| 108 | | Lawton, B. A., et al. (2008). "Exercise on prescription for women aged 40-74 recruited through primary care: two year randomised controlled trial." BMJ (Clinical research ed.) 337. |
| 109 | | Liira, H., et al. (2014). Exercise intervention and health checks for middle-aged men with elevated cardiovascular risk: A randomized controlled trial. Scandinavian Journal of Primary Health Care 32(4): 156-163. |
| 110 | | Lewin, R. J., et al. (2002) A randomised controlled trial of a self-management plan for patients with newly diagnosed angina. British Journal of General Practice 52, 194-196, 199-201 |
| 111 | | Lewis, B. S. and W. D. Lynch (1993) The effect of physician advice on exercise behavior. Preventive Medicine 22, 110-121 |
| 112 | | Little, P., et al. (2004) A randomised controlled trial of three pragmatic approaches to initiate increased physical activity in sedentary patients with risk factors for cardiovascular disease. British Journal of General Practice 54, 189-195 |
| 113 | | Logghe, I. H., et al. (2009) Lack of effect of Tai Chi Chuan in preventing falls in elderly people living at home: a randomized clinical trial. Journal of the American Geriatrics Society 57, 70-75 DOI: 10.1111/j.1532-5415.2008.02064.x |
| 114 | | Logue, E., et al. (2005) Transtheoretical model-chronic disease care for obesity in primary care: a randomized trial. Obesity research 13, 917-927 DOI: 10.1038/oby.2005.106 |
| 115 | | Margareta Eriksson, K., et al. (2006) A randomized trial of lifestyle intervention in primary healthcare for the modification of cardiovascular risk factors. The Bjorknas study. Scandinavian Journal of Public Health 34, 453-461 DOI: 10.1080/14034940500489826 |
| 116 | | Marshall, A. L., et al. (2005) Promoting physical activity in Australian general practices: a randomised trial of health promotion advice versus hypertension management. Patient Education and Counseling 56, 283-290 DOI: 10.1016/j.pec.2004.03.002 |
| 117 | | McMurdo, M. E. T., et al. (2010). "Do pedometers increase physical activity in sedentary older women? A randomized controlled trial." Journal of the American Geriatrics Society 58(11): 2099-2106. |
| 118 | | Mehring, M., et al. (2013). "Effects of a general practice guided web-based weight reduction program - results of a cluster-randomized controlled trial." BMC Family Practice 14(76): 8. |
| 119 | | Migneault, J. P., et al. (2012). "A culturally adapted telecommunication system to improve physical activity, diet quality, and medication adherence among hypertensive African-Americans: a randomized controlled trial." Annals of behavioral medicine : a publication of the Society of Behavioral Medicine 43(1): 62-73. |
| 120 | | Moore, R. H., et al. (2013). "Changes in eating, physical activity and related behaviors in a primary care-based weight loss intervention." International Journal of Obesity 37(SUPPL.1): S12-S18. |
| 121 | | Morey, C., et al. (2012). "Enhanced Fitness: A Randomized Controlled Trial of the Effects of Home-Based Physical Activity Counseling on Glycemic Control in Older Adults with Prediabetes Mellitus." Journal of the American Geriatrics Society 60(9): 1655-1663. |
| 122 | | Morey, M. C., et al. (2006). "Project LIFE: a partnership to increase physical activity in elders with multiple chronic illnesses." Journal of Aging & Physical Activity 14(3): 324-344. |
| 123 | | Morey, M. C., et al. (2009). "The Veterans Learning to Improve Fitness and Function in Elders Study: a randomized trial of primary care-based physical activity counseling for older men." Journal of the American Geriatrics Society 57(7): 1166-1175. |
| 124 | | Muda, S. H. and A. A. Kadir (2006). "The effectiveness of physical activity counseling in Primary Care Clinic University Science Malaysia Hospital." International Medical Journal 13(4): 249-253. |
| 125 | | Mutrie, N., et al. (2012). "Increasing older adults' walking through primary care: Results of a pilot randomized controlled trial." Family Practice 29(6): 633-642. |
| 126 | | Niiranen, T. J., et al. (2014). "Lack of impact of a comprehensive intervention on hypertension in the primary care setting." American Journal of Hypertension 27(3): 489-496. |
| 127 | | Nilsen, V., et al. (2011) Effects of lifestyle intervention in persons at risk for type 2 diabetes mellitus - results from a randomised, controlled trial. BMC Public Health 11, 893 DOI: 10.1186/1471-2458-11-893 |
| 128 | | Pace, W. D., et al. (2013) Effectiveness of 2 methods of promoting physical activity, healthy eating, and emotional well-being with the americans in motion--healthy interventions approach. Annals of Family Medicine 11, 371-380 DOI: 10.1370/afm.1516 |
| 129 | | Parekh, S., et al. (2014). "Randomized controlled trial of a computer-tailored multiple health behaviour intervention in general practice: 12-month follow-up results." International Journal of Behavioral Nutrition and Physical Activity 11(1). |
| 130 | | Parra-Medina, D., et al. (2011). "Results of the Heart Healthy and Ethnically Relevant Lifestyle trial: a cardiovascular risk reduction intervention for African American women attending community health centers." American Journal of Public Health 101(10): 1914-1921. |
| 131 | | Patel, A., et al. (2013) The long-term effects of a primary care physical activity intervention on mental health in low-active, community-dwelling older adults. Aging & Mental Health 17, 766-772 |
| 132 | | Perry, C. K., et al. (2007) Heart-to-Heart: promoting walking in rural women through motivational interviewing and group support. Journal of cardiovascular nursing 22, 304-312 DOI: 10.1097/01.JCN.0000278953.67630.e3 |
| 133 | | Piette, J. D., et al. (2011). "A randomized trial of telephonic counseling plus walking for depressed diabetes patients." Medical Care 49(7): 641-649. |
| 134 | | Pinto, B. M., et al. (2005). "Randomized controlled trial of physical activity counseling for older primary care patients." American journal of preventive medicine 29(4): 247-255. |
| 135 | | Qi, B. B., et al. (2011) Self-efficacy program to prevent osteoporosis among Chinese immigrants: a randomized controlled trial. Nursing Research 60, 393-404 DOI: 10.1097/NNR.0b013e3182337dc3 |
| 136 | | Reed, J., et al. (2008). "An ecological approach with primary-care counseling to promote physical activity." Journal of physical activity & health 5(1): 169-183. |
| 137 | | Riley, K. M., et al. (2001) Resources for health: A social-ecological intervention for supporting self-management of chronic conditions. Journal of Health Psychology 6, 693-705 |
| 138 | | Rimmer, J. H., et al. (2009) A randomized controlled trial to increase physical activity and reduce obesity in a predominantly African American group of women with mobility disabilities and severe obesity. Preventive Medicine 48, 473-479 |
| 139 | | Rodríguez Martín, C., et al. (2009) [Efficacy of an educational intervention group on changes in lifestyles in hypertensive patients in primary care: a randomized clinical trial]. Revista Espanola de Salud Publica 83, 441-452 |
| 140 | | Rom, Å., et al. (2014). "Costs and outcomes of an exercise referral programme - A 1-year follow-up study." European Journal of Physiotherapy 16(2): 82-93. |
| 141 | | Rosenberg, D., et al. (2014) Integrated medical care management and behavioral risk factor reduction for multicondition patients: Behavioral outcomes of the TEAMcare trial. General hospital psychiatry 36, 129-134 DOI: http://dx.doi.org/10.1016/j.genhosppsych.2013.10.017 |
| 142 | | Ruffin, M. T., et al. (2011) Effect of preventive messages tailored to family history on health behaviors: the Family Healthware Impact Trial. Annals of Family Medicine 9, 3-11 DOI: 10.1370/afm.1197 |
| 143 | | Sakane, N., et al. (2011) Prevention of type 2 diabetes in a primary healthcare setting: three-year results of lifestyle intervention in Japanese subjects with impaired glucose tolerance. BMC Public Health 11, 40 DOI: 10.1186/1471-2458-11-40 |
| 144 | | Schousboe, J. T., et al. (2005) Education and phone follow-up in postmenopausal women at risk for osteoporosis: Effects on calcium intake, exercise frequency, and medication use. Disease Management and Health Outcomes 13, 395-404 DOI: 10.2165/00115677-200513060-00004 |
| 145 | | Shepich, J., et al. (2007) Do subsidization and monitoring enhance adherence to prescribed exercise? American Journal of Health Promotion 22, 2-5 |
| 146 | | Sniehotta, F. F., et al. (2011) Randomised controlled feasibility trial of an evidence-informed behavioural intervention for obese adults with additional risk factors. PLoS ONE 6, e23040 DOI: 10.1371/journal.pone.0023040 |
| 147 | | Sorensen, J. B., et al. (2008). "Exercise on prescription: A randomized study on the effect of counseling vs counseling and supervised exercise." Scandinavian Journal of Medicine and Science in Sports 18(3): 288-297. |
| 148 | | Staffileno, B. A., et al. (2007) Blood pressure responses to lifestyle physical activity among young, hypertension-prone African-American women. Journal of cardiovascular nursing 22, 107-117 |
| 149 | | Stevens, W., et al. (1998). "Cost-effectiveness of a primary care based physical activity intervention in 45-74 year old men and women: a randomised controlled trial... including commentary by Eaton CB." British Journal of Sports Medicine 32(3): 236-242. |
| 150 | | Stovitz, S. D., et al. (2005) Pedometers as a means to increase ambulatory activity for patients seen at a family medicine clinic. Journal of the American Board of Family Practice / American Board of Family Practice 18, 335-343 |
| 151 | | Stuart, K. L., et al. (2014) A telephone-supported cardiovascular lifestyle programme (CLIP) for lipid reduction and weight loss in general practice patients: a randomised controlled pilot trial. Public health nutrition 17, 640-647 DOI: 10.1017/S1368980013000220 |
| 152 | | Sugden, J. A., et al. (2008). "The feasibility of using pedometers and brief advice to increase activity in sedentary older women - A pilot study." BMC health services research 8. |
| 153 | | Swinburn, B. A., et al. (1998) The green prescription study: a randomized controlled trial of written exercise advice provided by general practitioners. American Journal of Public Health 88, 288-291 |
| 154 | | Tan, M. Y., et al. (2011) A brief structured education programme enhances self-care practices and improves glycaemic control in Malaysians with poorly controlled diabetes. Health Education Research 26, 896-907 DOI: 10.1093/her/cyr047 |
| 155 | | Taylor, A. H., et al. (1998) Randomised controlled trial to examine the effects of a GP exercise referral programme in Hailsham, East Sussex, on modifiable coronary heart disease risk factors. Journal of Epidemiology and Community Health 52, 595-601 |
| 156 | | ter Bogt, N. C., et al. (2011) Changes in lifestyle habits after counselling by nurse practitioners: 1-year results of the Groningen Overweight and Lifestyle study. Public health nutrition 14, 995-1000 DOI: 10.1017/S1368980010003708 |
| 157 | | Tiessen, A. H., et al. (2012) Randomized controlled trial on cardiovascular risk management by practice nurses supported by self-monitoring in primary care. BMC Family Practice 13, 90 DOI: 10.1186/1471-2296-13-90 |
| 158 | | Tokunaga-Nakawatase, Y., et al. (2014). Computer-supported indirect-form lifestyle-modification support program using Lifestyle Intervention Support Software for Diabetes Prevention (LISS-DP) for people with a family history of type 2 diabetes in a medical checkup setting: a randomized controlled trial. Primary Care Diabetes 8(3): 207. |
| 159 | | Toobert, D. J., et al. (2011) Outcomes from a multiple risk factor diabetes self-management trial for Latinas: ¡Viva Bien! Annals of Behavioral Medicine 41, 310-323 DOI: 10.1007/s12160-010-9256-7 |
| 160 | | Tully, M. A., et al. (2005) Brisk walking, fitness, and cardiovascular risk: a randomized controlled trial in primary care. Preventive Medicine 41, 622-628 DOI: 10.1016/j.ypmed.2004.11.030 |
| 161 | | Van, I. W., et al. (2012). "Effectiveness of peer-led self-management coaching for patients recently diagnosed with Type 2 diabetes mellitus in primary care: A randomized controlled trial." Diabetic Medicine 29(10): e390-e397. |
| 162 | | Vermunt, P. W., et al. (2012) A lifestyle intervention to reduce Type 2 diabetes risk in Dutch primary care: 2.5-year results of a randomized controlled trial. Diabetic Medicine 29, e223-231 DOI: 10.1111/j.1464-5491.2012.03648.x |
| 163 | | Voils, C. I., et al. (2013). "A randomized controlled trial to evaluate the effectiveness of CouPLES: A spouse-assisted lifestyle change intervention to improve low-density lipoprotein cholesterol." Preventive Medicine 56(1): 46-53. |
| 164 | | Voogdt-Pruis, H. R., et al. (2011) Adherence to a guideline on cardiovascular prevention: a comparison between general practitioners and practice nurses. International Journal of Nursing Studies 48, 798-807 DOI: 10.1016/j.ijnurstu.2010.11.008 |
| 165 | | Vrdoljak, D., et al. (2014). "Lifestyle intervention in general practice for physical activity, smoking, alcohol consumption and diet in elderly: A randomized controlled trial." Archives of Gerontology and Geriatrics 58(1): 160-169. |
| 166 | | Wadden, T. A., et al. (2009) One-year weight losses in the Look AHEAD study: factors associated with success. Obesity (Silver Spring, Md.) 17, 713-722 DOI: 10.1038/oby.2008.637 |
| 167 | | Warren, F. C., et al. (2014). "Evaluation of different recruitment and randomisation methods in a trial of general practitioner-led interventions to increase physical activity: A randomised controlled feasibility study with factorial design." Trials 15(1). |
| 168 | | Weinstock, R. S., et al. (2011). "Lessened decline in physical activity and impairment of older adults with diabetes with telemedicine and pedometer use: Results from the IDEATel study." Age and Ageing 40(1): 98-105. |
| 169 | | Whitehead, D., et al. (2007). "A stage-targeted physical activity intervention among a predominantly African-American low-income primary care population." American Journal of Health Promotion 21(3): 160-164. |
| 170 | | Whittemore, R., et al. (2009). "Translating the diabetes prevention program to primary care: a pilot study." Nursing Research 58(1): 2-13. |
| 171 | | Worm, C. H., et al. (2001) Effects of a multicomponent exercise program on functional ability in community-dwelling, frail older adults. Journal of Aging and Physical Activity 9, 414-424 |
| **RCTs with no information on PROGRESS-Plus** | | |
| 172 | Drevenhorn, E., et al. (2012) Consultation training of nurses for cardiovascular prevention - a randomized study of 2 years duration. Blood pressure 21, 293-299 DOI: 10.3109/08037051.2012.680734 | |
| 173 | Faulkner, J., et al. (2010). "The feasibility of recruiting patients with early COPD to a pilot trial assessing the effects of a physical activity intervention." Primary Care Respiratory Journal 19(2): 124-130. | |
